# Posterior longitudinal strain by speckle tracking echocardiography, marker of cardiac amyloidosis?

**DOI:** 10.1186/1750-1172-10-S1-P48

**Published:** 2015-11-02

**Authors:** Julien Ternacle, Jean-François Deux, Diane Bodez, Aziz Guellich, Alain Rahmouni, Jean-Luc Dubois-Randé, Violaine Plante-Bordeneuve, Thibaud Damy

**Affiliations:** 1 CHU H Mondor, Amyloidosis Mondor Creteil, 94000, Créteil, France

## Background

Cardiac amyloidosis (CA) is a condition of poor prognosis. The three major forms of amyloidosis are light chain (AL), hereditary transthyretin (M-TTR), and wild-type transthyretin (WT-TTR). Two-dimensional (2D) echocardiography measurement of longitudinal strain (LS) has been reported to be useful in the diagnosis of CA. Regional distribution of LS in CA and its diagnostic value in detecting early left-ventricular systolic dysfunction is unclear.

## Objectives

To compare left ventricular LS evaluated by 2D echocardiography with cardiac magnetic resonance imaging (MRI) in CA.

## Methods

Patients with cardiac amyloidosis were included prospectively. Inclusion criteria were age >18 years, diagnosis of amyloidosis with cardiac involvement defined by an interventricular septum wall thickness (IVST) above 12 mm. For each of the 17 left-ventricular segments in the American Heart Association model, we evaluated LS and late gadolinium enhancement (LGE) by MRI.

## Results

Among the 162 patients with amyloidosis, 97 had CA and were included in the study; 30 had AL, 46 m-TTR, and 21 WT-TTR. Mean LS was -11±4% and was similarly impaired in the three types of amyloidosis. 69 patients had cardiac MRI of whom 64 (93%) had positive LGE. The number of segments with LGE was similar across the three CA types. All the 69 patients had basal posterior wall involvement as reflected by decreased LS (-6±6%). Both LS and amyloid deposits showed a basal-to-apical gradient. A significant correlation was found between basal posterior wall LS and the number of segments with LGE (r=0.56, p<0.001).

## Conclusions

Basal-to-apical LS abnormalities are similar across CA types. Basal posterior wall LS may be used to appreciate the severity of cardiac amyloidosis.

